# Avian Malaria and Related Parasites from Resident and Migratory Birds in the Brazilian Atlantic Forest, with Description of a New *Haemoproteus* Species

**DOI:** 10.3390/pathogens10020103

**Published:** 2021-01-21

**Authors:** Carolina C. Anjos, Carolina R. F. Chagas, Alan Fecchio, Fabio Schunck, Maria J. Costa-Nascimento, Eliana F. Monteiro, Bruno S. Mathias, Jeffrey A. Bell, Lilian O. Guimarães, Kiba J. M. Comiche, Gediminas Valkiūnas, Karin Kirchgatter

**Affiliations:** 1 Programa de Pós-Graduação em Medicina Tropical, Instituto de Medicina Tropical, Faculdade de Medicina, Universidade de São Paulo, São Paulo 05403-000, SP, Brazil; carolinaclares@gmail.com (C.C.A.); elianafmonteiro@usp.br (E.F.M.); brunomathiasbio@gmail.com (B.S.M.); kibajamila@hotmail.com (K.J.M.C.); 2 Nature Research Centre, 08412 Vilnius, Lithuania; crfchagas@gmail.com (C.R.F.C.); gediminas.valkiunas@gamtc.lt (G.V.); 3 Programa de Pós-graduação em Ecologia e Conservação da Biodiversidade, Universidade Federal de Mato Grosso, Cuiabá 78060-900, Brazil; alanfecchio@gmail.com; 4 Comitê Brasileiro de Registros Ornitológicos—CBRO, São Paulo 04785-040, SP, Brazil; fabio_schunck@yahoo.com.br; 5 Núcleo de Estudos em Malária, Superintendência de Controle de Endemias, Instituto de Medicina Tropical, Faculdade de Medicina, Universidade de São Paulo, São Paulo 05403-000, SP, Brazil; dida.sucen@gmail.com; 6 Department of Biology, University of North Dakota, 10 Cornell Street, Grand Forks, ND 58202, USA; jeffrey.bell@und.edu; 7 Laboratório de Bioquímica e Biologia Molecular, Superintendência de Controle de Endemias, São Paulo 01027-000, SP, Brazil; lilianguima@gmail.com

**Keywords:** avian migration, avian malaria, *Plasmodium*, *Haemoproteus*, parasite diversity, phylogenetic diversity, vector borne disease

## Abstract

Determining the prevalence and local transmission dynamics of parasitic organisms are necessary to understand the ability of parasites to persist in host populations and disperse across regions, yet local transmission dynamics, diversity, and distribution of haemosporidian parasites remain poorly understood. We studied the prevalence, diversity, and distributions of avian haemosporidian parasites of the genera *Plasmodium*, *Haemoproteus*, and *Leucocytozoon* among resident and migratory birds in Serra do Mar, Brazil. Using 399 blood samples from 66 Atlantic Forest bird species, we determined the prevalence and molecular diversity of these pathogens across avian host species and described a new species of *Haemoproteus*. Our molecular and morphological study also revealed that migratory species were infected more than residents. However, vector infective stages (gametocytes) of *Leucocytozoon* spp., the most prevalent parasites found in the most abundant migrating host species in Serra do Mar (*Elaenia albiceps*), were not seen in blood films of local birds suggesting that this long-distance Austral migrant can disperse *Leucocytozoon* parasite lineages from Patagonia to the Atlantic Forest, but lineage sharing among resident species and local transmission cannot occur in this part of Brazil. Our study demonstrates that migratory species may harbor a higher diversity and prevalence of parasites than resident species, but transportation of some parasites by migratory hosts may not always affect local transmission.

## 1. Introduction

Brazil is one of the world’s richest countries in terms of bird species diversity, with 1919 known species [1], of which 234 species are endangered [2]. In Brazil, the Atlantic Forest biome has the second largest number of species (934) and second highest level of endemism (18%) [3,4]. Although, the majority of Brazilian birds are resident species, 198 species are migratory [5]. Serra do Mar stands out as an important area of South American endemism and priority for the conservation of endemic and threatened species of this biome [6]. The largest area of preserved Atlantic Forest in Brazil is the Parque Estadual Serra do Mar (PESM) in the State of São Paulo. PESM is a forest reserve created in 1977 that currently encompasses 332,290 hectares divided into ten independent administrative centers (subregions named “núcleos”) [7]. Núcleo Curucutu covers an area of 36,134 ha inside this park and is one of the best known ornithologically regions in Southeast Brazil containing 382 bird species. A total of 124 bird species endemic to the Atlantic Forest have been recorded in this site, 38 of which are migratory, and 24 that are threatened with extinction. This area hosts migratory birds from different American countries and other Brazilian regions [8].

Parasitism has a strong influence on the dynamics and structure of biological communities [9], and can favor the decline of birds’ clinical conditions and decrease their ability to survive and reproduce, which can affect population size and even cause extinctions [10]. Malaria is an infectious disease, caused by blood protists belonging to the genus *Plasmodium* (Apicomplexa: Haemosporida), a cosmopolitan group of heteroxenous protists that parasitize amphibians, reptiles, birds, and mammals and are transmitted by mosquitoes (Culicidae) [11,12]. *Haemoproteus* and *Leucocytozoon* species are closely related to malaria parasites with worldwide distributions, and they are commonly found in birds [12]. *Haemoproteus* spp. are transmitted by biting midges (Ceratopogonidae) and hippoboscid flies (Hippoboscidade). Whereas, *Leucocytozoon* parasites are mainly transmitted by black flies (Simuliidae), with one species transmitted by midges (Ceratopogonidae) [12]. The study of avian haemosporidian parasites is important due to their ecological and conservation aspects, since their presence in birds can influence their hosts’ ecological, evolutionary, and behavioral processes, including flight modifications, reproductive success, clutch size, migration, competition, and foraging capacity (revision in [13]). Moreover, the spread of parasites via bird migration may help drive the worldwide distribution of avian haemosporidian parasites [14]. Therefore, the aim of this study is the detection of haemosporidian parasites, through molecular and microscopic techniques, in resident and migratory bird species from the Brazilian Atlantic Rainforest to understand the role of migration in distribution and diversity of these parasites. Additionally, we describe of a new species of *Haemoproteus*.

## 2. Results 

**Figure 1:**
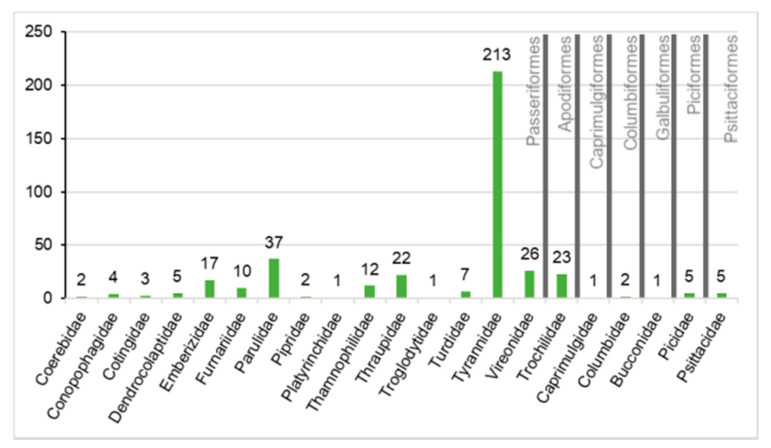
Absolute numbers of the examined birds according to order and family studied.

We surveyed haemosporidian infections in blood samples collected from 399 birds, belonging to 66 species, 21 families, and seven orders (Apodiformes, Caprimulgiformes, Columbiformes, Galbuliformes, Passeriformes, Piciformes, Psittaciformes). Species of Passeriformes represented 90.7% of all analysed samples. Species of Apodiformes (5.8%), Columbiformes (0.5%), Piciformes (1.25%), and Psittaciformes (1.25%) were less frequently sampled and together represented only 8.8% of analysed samples. A third group (rarely sampled birds) contained representatives of two different orders: Caprimulgiformes and Galbuliformes (0.5% of all analysed samples) (Figure 1). 

### 2.1. Microscopic Examination

We collected 195 thin blood smears in 2019 from 135 bird samples. Haemosporidian parasites were only identified by microscopy in 11 birds: two were infected with *Plasmodium nucleophilum* (identified by the nucleophilic pattern of blood stages and the presence of all the other main characteristics of this species (see [15]), three with *Plasmodium* sp., four with *Haemoproteus* (*Parahaemoproteus*) sp., one with mixed infection (*Plasmodium* sp. + *Haemoproteus* sp.), and one with *Haemoproteus* (*Parahaemoproteus*) *nucleocentralis* n. sp. which is described below. 

**Figure 2:**
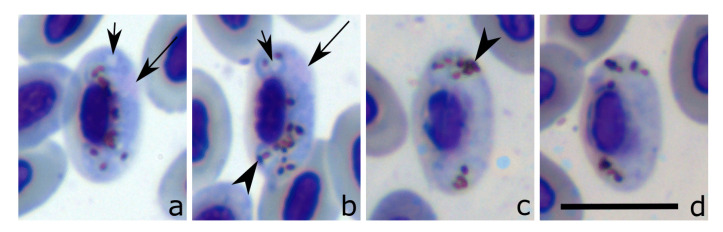
*Haemoproteus* sp. (cytochrome *b* lineage hELAALB01) from *Elaenia albiceps*. (**a–b**): macrogametocytes. (**c–d**): microgametocytes. Note the presence of a vacuole (**a–b**). Long arrows: gametocyte nuclei; arrowheads: pigment granules; short arrow: vacuole. Giemsa-stained thin blood films. Scale bar: 10 μm.

Among the *Plasmodium*-infected individuals for which we analysed blood smears, three were from resident host species (*Tachyphonus coronatus*, *Turdus rufiventris,* and *Zonotrichia capensis*) and three from one migratory species (*Elaenia albiceps*). We found *Plasmodium* gametocytes in the blood smears of one Curucutu resident bird (*Zonotrichia capensis*) and two migratory birds (*Elaenia albiceps*). In contrast, among the *Haemoproteus*-infected individuals for which we analysed blood smears, one was a resident species (*Tangara desmaresti*) and four were migratory species (*Elaenia albiceps*). The *Haemoproteus* parasite found in the *E. albiceps* is a new lineage (see discussion below) and likely a new species, but, due to the poor quality of staining, it was not possible to describe this species. Even with poor staining, it was possible to notice some distinctive morphological features of this parasite, such as the presence of a readily visible vacuole in the macrogametocytes (Figure 2a,b), sub-terminal position of nuclei in macrogametocytes (Figure 2a,b), close adherence of advanced gametocytes, both to the nuclei and envelope of erythrocytes (Figure 2a–d), and slight displacement of nuclei in infected erythrocytes (Figure 2a–d). For further parasite description, additional material is needed. 

#### 2.1.1. New Parasite Description

Family Haemoproteidae Doflein, 1916

Genus *Haemoproteus* Kruse, 1890

Haemoproteus (Parahaemoproteus) nucleocentralis n. sp.

Type host: Brassy-breasted Tanager *Tangara desmaresti* (Vieillot, 1819) (Passeriformes, Thraupidae).

Type locality: Núcleo Curucutu, Parque Estadual Serra do Mar, São Paulo, SP (23°85′60″ S, 46°83′90″ W, 800 m a.s.l.), Brazil.

Type specimens: Hapantotypes (accession numbers PESM610A and PESM610B, juvenile, male, *Tangara desmaresti* UFMT 5063; parasitaemia 0.1%, 9.iii.2019, Núcleo Curucutu, Parque Estadual Serra do Mar, São Paulo, Brazil, collected by F. Schunck) were deposited in the Universidade Federal de Mato Grosso, Brazil. 

Site of infection: Mature erythrocytes; no other data.

Prevalence: one examined Brassy-breasted Tanager was infected.

Representative DNA sequence: Mitochondrial *cytb* lineage hTANDES01 (478 bp, GenBank accession number MT724553).

Vector: Probably *Culicoides* biting midges; species is unknown (see Discussion).

Etymology: The species name refers to a distinctive character of this species, which is the predominantly central position of nuclei in fully-grown macrogametocytes (see Description below).

##### Description 

**Figure 3:**
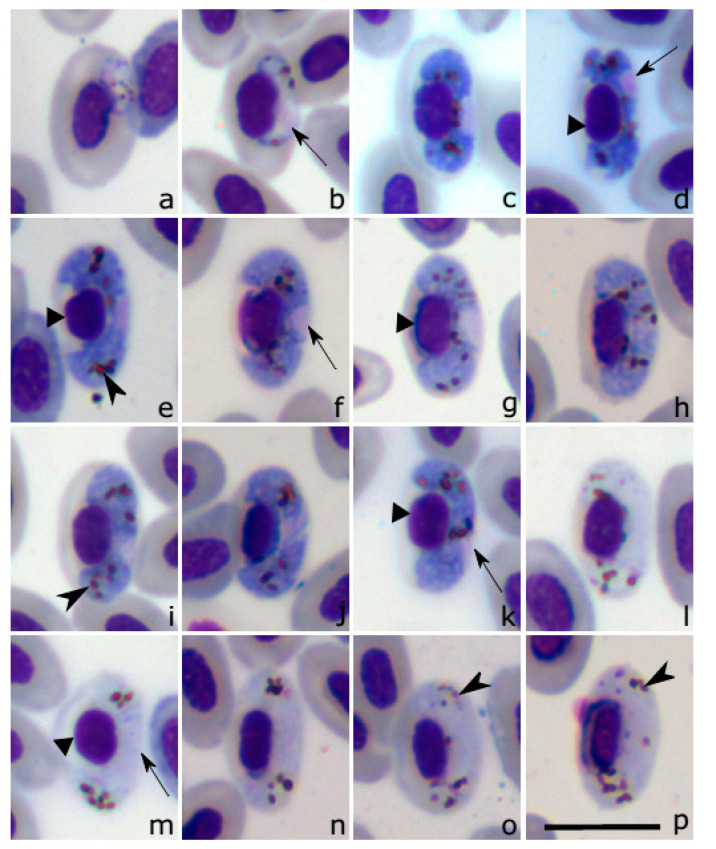
*Haemoproteus (Parahaemoproteus) nucleocentralis* n. sp. (cytochrome *b* lineage hTANDES01) from *Tangara desmaresti*. (**a**) Young gametocyte. (**b**) Growing gametocyte. (**c–k**) macrogametocytes. (**l–p**) Microgametocytes. Note that nuclei were located in central or close to central position in all mature macrogametocytes (**e–k**), but they were occasionally seen in sub-terminal position in the growing parasites (**d**). Long arrows: Gametocyte nuclei; arrowheads: Pigment granules; triangle arrowhead: Nuclei of infected erythrocytes. Giemsa-stained thin blood films. Scale bar: 10 μm.

Young gametocytes (Figure 3a,b) were rare in the type material; they develop in mature erythrocytes. Earliest gametocytes are broadly oval in form; they adhere to the nuclei of erythrocytes and were seen in a subpolar position in infected erythrocytes (Figure 3a); pigment granules were readily visible. Advanced growing gametocytes closely adhere both to the erythrocyte nuclei and envelope; they extend longitudinally along the nuclei and slightly displace them laterally (Figure 3b). Gametocyte outline is even. Pigment granules are prominent; some of them reach size of pigment granules present in mature gametocytes (compare Figure 3b and Figure 3g). 

Macrogametocytes (Figure 3c–k) develop in mature erythrocytes. The cytoplasm is homogeneous or slightly granular in appearance. Volutin granules and vacuoles were not seen. Outline is predominantly even (Figure 3e–g), occasionally slightly wavy at gametocyte ends (Figure 3d,e). Gametocytes grow along nuclei of infected erythrocytes, enclose nuclei with their ends, but do not encircle them completely (Figure 3c–k). Advanced and fully-grown macrogametocytes were closely appressed both to the envelop and nuclei of host cells; they displace the nuclei laterally (Table 1) and slightly deform them, resulting in acceptance of a slightly roundish shape in comparison to nuclei in non-infected erythrocytes (Figure 3c,e,k). Fully-grown gametocytes fill erythrocytes up to their poles (Figure 3e–k). Gametocytes nucleus is relatively small (Table 1), variable in form, predominantly assumes a central or close to the central position (Figure 3d–g), a characteristic feature of this species development; occasionally the nuclei were observed in slightly sub-central position (Figure 3d,i,k), nucleolus was not seen. Pigment granules can be scattered throughout the cytoplasm (Figure 3g), but also often seen in groups (Figure 3c–f). Pigment granules are variable in form and shape, they usually are oval or slightly elongate; predominate medium-size granules (0.5–1.0 μm), but a few of large-size (1.0–1.5 µm) granules were often seen. Fully-grown gametocytes displace nuclei of host-cells laterally, but do not influence shape of the cells in comparison to uninfected erythrocytes (Figure 3e–h, Table 1).

Microgametocytes (Figure 3l–p, Table 1). General configuration and other characters are as in macrogametocytes with the usual haemosporidian sexual dimorphic characters, which are the large diffuse nuclei and relatively pale staining of the cytoplasm. 

##### Taxonomic remarks

*Haemoproteus nucleocentralis* n. sp. is the first haemosporidian parasite reported in *Tangara desmaresti*. The most similar partial *cytb* sequences (GenBank accessions MN459077 and KJ466075) are of 99% similarity (or 2 bp); they were reported in the Orange-bellied Euphonia *Euphonia xanthogaster* (Passeriformes, Thraupidae) in the Chilean Andes. However, there is no morphological characterization of these parasites and no publication associated with these DNA sequences. In the MalAvi database, the closest lineage to the new species is hOCHLEU01, with 98% similarity (or 5 bp); it was reported in several species of Thraupidae in South American Andean birds.

*Haemoproteus coatneyi* was often reported in birds belonging to the *Tangara* genus and other Thraupidae birds [17], thus, should be distinguished from the new species. A characteristic feature of *H. nucleocentralis* n. sp. is the predominantly central position of nuclei in fully-grown gametocytes. This character is relatively rare in haemoproteids parasitizing passerine birds and has not been reported in haemoproteids parasitizing New World passerines as of yet, so is worthy of attention during *H. nucleocentralis* identification. Based on this character, gametocytes of *H. nucleocentralis* can be readily distinguished from *Haemoproteus coatneyi* [18], the common parasite of New World passerines. In *H. coatneyi,* nuclei are strictly sub-terminal in macrogametocytes [12]. The same is seen in, *Haemoproteus paruli* [18] and *Haemoproteus thraupi* [18], which have gametocytes morphologically indistinguishable from *H. coatneyi* [12]. Another distinctive feature of *H. nucleocentralis* n. sp. is the deformed infected host-cell nuclei, which assume a roundish form (compare Figure 3a,b with Figure 3e,k), but this feature remains insufficiently investigated in the other above-mentioned *Haemoproteus* parasites. 

*Haemoproteus nucleocentralis* n. sp. can be readily distinguished from *Haemoproteus erythrogravidus*, a common parasite of New World passerines, due to the absence of protrusion in the envelope of the infected erythrocyte (so-called the gravid morphology of infected host cells) [19]. Additionally, nuclei located strictly in sub-terminal position in macrogametocytes of *H. erythrogravidus,* and this is not the case in the new species.

Gametocytes of *H. nucleocentralis* n. sp. share some similar features with *Haemoproteus witti*, a common parasite of hummingbirds (Apodiformes), in the New World. In both species, nuclei are predominantly of central position in macrogametocytes [20]. Interestingly, several lineages of *H. witti* were reported in passerines, but gametocytes were not, indicating possible incomplete (abortive) development of this hummingbird parasite in passerines. In *H. witti* gametocytes, the average number of pigment granules is close to 25, which is significantly less than (about 10) in *H. nucleocentralis* (Table 1). 

*Haemoproteus nucleocentralis* n. sp. can be readily distinguished from *Haemoproteus vireonis,* a common parasite of South American passerines [12,21]. In the latter parasites, the growing gametocytes often assume the dumbbell-shape and nuclei locate strictly sub-terminally in macrogametocytes. Both these features are not characteristics of *H. nucleocentralis* n. sp. 

Phylogenetic inference showed that cytb sequences of H. nucleocentralis n. sp. clustered with Haemoproteus paruli (see 2.3. Molecular and Phylogenetic Analysis). These two lineages are only of 96% similarity (21 bp difference) in partial cytb sequences. Gametocytes of the latter parasite are indistinguishable morphologically from those of H. coatneyi [12]. Cytb sequences of H. coatneyi and H. paruli are available, and are located in different branches in the phylogenetic tree. However, it should be noted that morphological data were not provided when linking these sequences with corresponding morphospecies [22], and the accuracy of the molecular characterization of both H. coatneyi and H. paruli needs further support.

### 2.2. Infection Prevalence in Resident and Migratory Avifauna

From the 399 samples, 181 were from 52 resident avian species, and 218 samples were from 14 migratory species (Table 2; Table 3). All the 14 migratory species were from the order Passeriformes (Table 3). *Elaenia albiceps* were particularly extensively sampled, comprising 37.6% of all collected samples. 

Prevalence differed significantly between resident birds, 16 infections (9% prevalence), and migratory birds, 52 infections (24% prevalence) (X^2^ (1, *N* = 399) = 15.77, *p* = 0.00007), mainly due to *Leucocytozoon* spp.: (X^2^ (1, *N* = 399) = (Yates’ correction) 15.61, *p* = 0.00008) and *Haemoproteus* spp.: (X^2^ (1, *N* = 399) = (Yates’ correction) 4.40, *p* = 0.036); but not for *Plasmodium* spp.: (X^2^ (1, *N* = 399) = 0.76, *p* = 0.38) ( Table 2; Table 3). Prevalence remained significant after considering only Passeriformes (X^2^ (1, *N* = 362) = 10.38, *p* = 0.0013) or when removing *Elaenia albiceps* (X^2^ (1, *N* = 212) = 5.15, *p* = 0.023). *Elaenia albiceps* had the highest infection prevalence (24.7% prevalence within this species, which includes 71.1% of all infections within migratory birds and 54.4% of total infections identified). 

Except for the lineages with widespread (global) transmission (pCOLL4, pDENPET03, pPADOM09, pPADOM11, pTUMIG03), lineages likely transmitted predominantly in South America (pTRMEL02, hELAALB01, hVIGIL09, hVIOLI05, hCHIPAR01, lDIUDIU11, lELAALB02, lELAALB05, lZOLPYR01, lTROAED02) were found exclusively in migratory species and lineages transmitted in Brazil (pCONLIN16, pLEAMA01, pPYLEU01, pSPMAG06, pTARUF01) were mostly found infecting exclusively resident birds ( Table 2; Table 3; Supplementary Table S1). 

### 2.3. Molecular and Phylogenetic Analysis

All samples were analysed by nested PCR and subsequent sequencing to detect the presence of *Plasmodium*, *Haemoproteus,* and *Leucocytozoon*. Among these, 68 were positive: 48.5% (33) for *Plasmodium*, 23.5% (16) for *Haemoproteus,* and 26.5% (18) for *Leucocytozoon*, in addition to a mixed infection with *Plasmodium* and *Haemoproteus* (1.5%).

We obtained sequences from 66 of the 68 infections detected, which were assigned to 31 haemosporidian lineages based on nucleotide sequences of the parasite’s *cytb* gene (Supplementary Table S1). In two *Leucocytozoon* infections (one from the 2017 collection and one from the 2019 collection), more than one *Leucocytozoon* lineage was found, making it impossible to determine the sequences without cloning procedures. Nineteen of the recognized lineages were *Plasmodium*, seven were *Haemoproteus*, and the remaining five were *Leucocytozoon* (Table 2; Table 3). The lineages obtained for *Plasmodium* and *Haemoproteus* (except for a hummingbird sample) are part of a large study of avian malaria in the Brazilian Atlantic Forest region [23], GenBank #MT724397-MT724400, MT724468-MT724472, MT724527-MT724567). All the *Leucocytozoon* sequences and the sequence obtained to a hummingbird sample were submitted to GenBank database exclusively for this study (Accession numbers #MW394193-MW394211). 

From the total lineages obtained, twenty (pBASCUL01, pCOLL4, pCONLIN16, pCURCUR01, pELAALB07, pGEOTRI01, pLEAMA01, pPADOM11, pPHPAT01, pPYLEU01, pRAMCAR05, pSPMAG06, pTARUF01, pTRMEL02, pTUMIG03, hCHIPAR01, hTANDES01, hVIOLI05, hVIGIL09, and lZOLPYR01) were found only once in the current study. The lineages pDENPET03 (*N* = 8), hELAALB01 (*N* = 8), lELAALB05 (*N* = 7), pVIOLI03 (*N* = 6), lDIUDIU11 (*N* = 4), lELAALB02 (*N* = 4), pPADOM09 (*N* = 3), hMYISWA01 (*N* = 3), pLEPCOR05 (*N* = 2), hZOCAP01 (*N* = 2), and lTROAED02 (*N* = 2) were found in more than one individual.

Although *Plasmodium* lineages pBASCUL01, pCONLIN16, pCURCUR01, pGEOTRI01, pLEAMA01, pPYLEU01, pSPMAG06, pTARUF01, pTUMIG03, and *Haemoproteus* lineages hTANDES01 and hZOCAP01, were found only in resident host species, *Plasmodium* lineages pDENPET03 (*N* = 8), pLEPCOR05 (*N* = 2), and pVIOLI03 (*N* = 6), were found in both migratory and resident birds (Table 2; Table 3). Among the 20 lineages found in migratory birds, ten were *Plasmodium*, five *Haemoproteus*, and five *Leucocytozoon*. 

Of the 19 *Plasmodium,* seven *Haemoproteus,* and five *Leucocytozoon* lineages found, 29 had already been reported and three are new reports (pBASCUL01, pELAALB07, hTANDES01) (Supplementary Table S1)

The lineage pBASCUL01 was found in *Basileuterus culicivorus* and hTANDES01 was reported here in *Tangara desmaresti.* This last one represents the first description of haemosporidian parasite in this species of passerines.

*Elaenia albiceps* had specimens infected by lineages that are widely transmitted, pDENPET03 (Argentina, Brazil, Canada, Guiana, Peru, Uruguay, and USA), others restricted to South America, pLEPCOR05 (Brazil and Peru), and even lineages described only in Argentina, (hELAALB01) (Table 2 and Supplementary Table S1).

**Figure 4:**
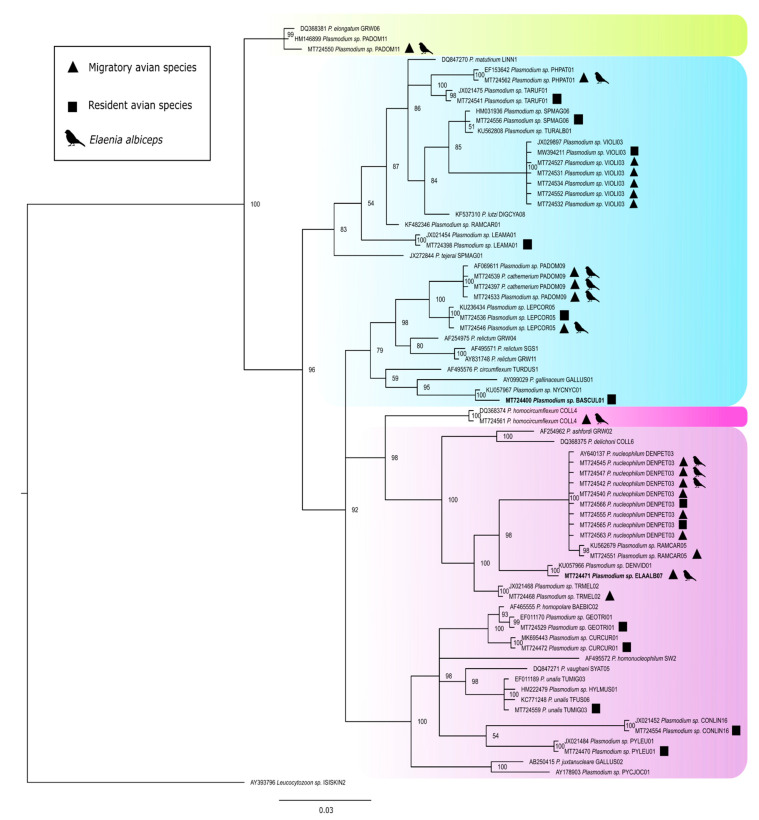
Bayesian phylogeny based on the partial *cytb* mitochondrial gene sequences of *Plasmodium* species. *Leucocytozoon* sp. was used as the outgroup. The support values of the nodes (in percentage) indicate posterior probabilities. The yellow box indicates the lineages and species of the subgenus *Huffia*, the blue box indicates the lineages and species of the subgenus *Haemamoeba*, the light pink box indicates the lineages and species of the subgenus *Novyella,* and the dark pink indicates the lineages and species of the subgenus *Giovannolaia*. The lineages reported during the present study were identified by the symbols “triangle” and “square”, for migratory and resident species, respectively. Lineages given in bold refer to new lineages.

Based on the *Plasmodium* lineage phylogenetic analysis (Figure 4), it is likely that most of the lineages found in Núcleo Curucutu belong to the subgenera *Haemamoeba* and *Novyella*. According to the MalAvi Database, the pDENPET03 (*P. nucleophilum*) lineage has already been described in 68 hosts belonging to 59 genera, 23 families, and eight orders in North and South America. In this study, the lineage was found in eight individuals of five species, belonging to five families, all of which are passerines. Although not statistically significant as mentioned above, it is also important to note a trend of higher occurrence of *Plasmodium* sp. in migratory species than in resident birds (18.6% versus 15.4%) (Supplementary Materials, Raw Data Spreadsheet S2).

**Figure 5:**
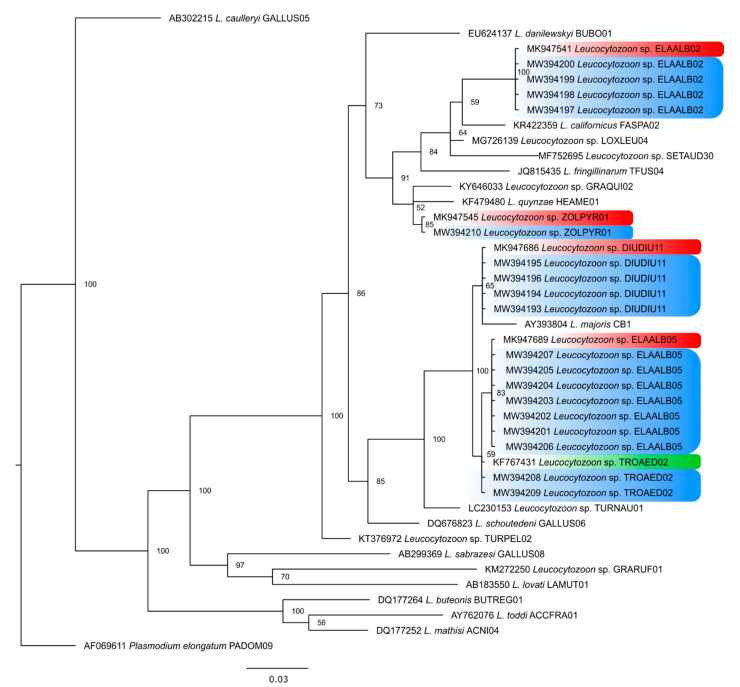
Bayesian phylogeny based on the partial *cytb* mitochondrial gene sequences of *Leucocytozoon* species. *Plasmodium elongatum* was used as the outgroup. Node support values (in percentage) indicate posterior probabilities. In blue the position of the lineages found in this study. In red, lineages described in Argentina, and in green, lineage described in Colombia.

All the 20 positive samples for *Leucocytozoon* were detected by PCR in *Elaenia albiceps*. Of these, 18 unique DNA sequences were obtained, since two contained overlapping *Leucocytozoon* sequences. Two of these 18 included mixed infections: one with *Plasmodium* sp. and another with *P. nucleophilum*. Five different lineages were obtained (lDIUDIU11, lELAALB02, lELAALB05, lZOLPYR01, and lTROAED02) with previous records in Argentina, Chile, Colombia, and Peru, also in *Elaenia albiceps* or *Elaenia frantzii* (Figure 5, Table 2 and Supplementary Table S1). 

**Figure 6:**
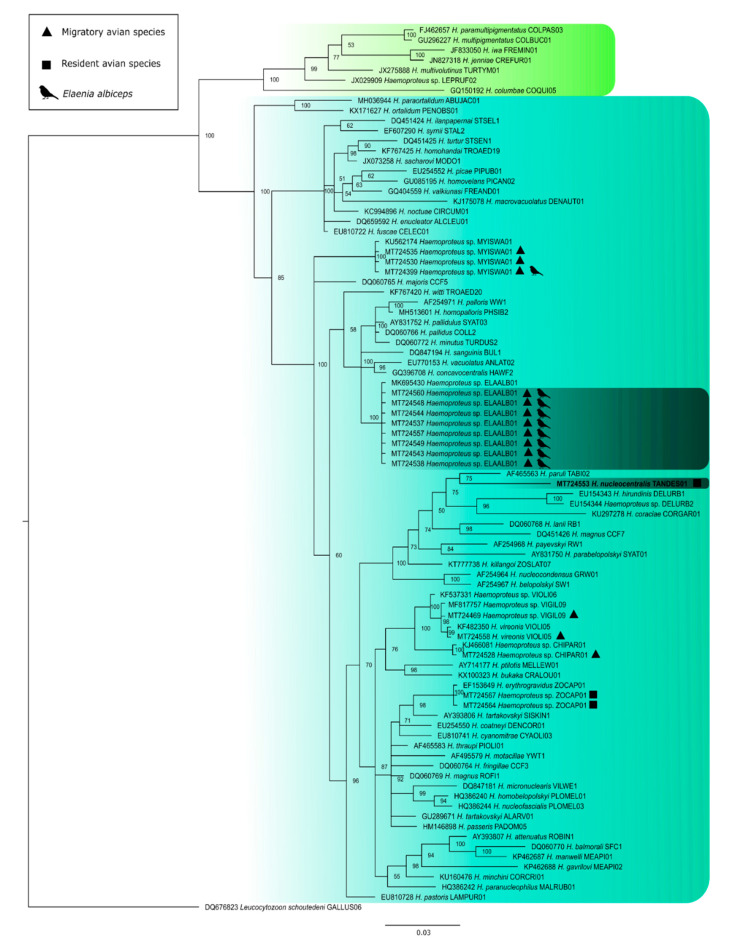
Bayesian phylogeny based on the partial *cytb* mitochondrial gene sequences of *Haemoproteus* species. *Leucocytozoon schoutedeni* was used as the outgroup. Node support values (in percentage) indicate posterior probabilities. In color the two subgenera *Parahaemoproteus* and *Haemoproteus*. The lineages found in the study are shown (represented with the symbols characterizing the species as resident and migratory). *Haemoproteus (Parahaemoproteus) nucleocentralis* n. sp. (hTANDES01 lineage) is given in bold. The lineages whose species are described morphologically are illuminated in dark green.

The Bayesian phylogeny based on the *cytb* gene of *Haemoproteus* species demonstrates the high prevalence of *Haemoproteus* sp. in migratory birds, as 14 of 17 infected individuals were detected in migratory avifauna [X^2^ (1, *N* = 399) = (Yates’ correction) 4.40, *p* = 0.036] (Figure 6) (Supplementary Materials, Raw Data Spreadsheet S2). The hELAALB01 lineage had the highest number of occurrences, with eight individuals of the migratory species *Elaenia albiceps* infected (Figure 6). The hELAALB01 lineage shows 99% identity with the lineages hPHSIB2 (*H. homopalloris*), hCOLL2 (*H. pallidus*), and hSYAT03 (*H. pallidulus*), and exist within a larger clade within the phylogenetic tree (Figure 6). All these species are known to have pale staining gametocytes. Moreover, the new hTANDES01 lineage described here as a new species (*H. nucleocentralis)* appeared in the clade of haemoproteids belonging to subgenus *Parahaemoproteus*, indicating that species of *Culicoides* (Ceratopogonidae) are involved in its transmission.

## 3. Discussion

We analysed blood samples from birds from Núcleo Curucutu belonging to seven orders, 21 families, and 66 species, the majority belonging to the family Tyrannidae (53.4%). A great diversity of haemosporidian parasites was found, including *Plasmodium* and *Haemoproteus* lineages widely described from passerines in the Neotropics and Europe (MalAvi database) and the first molecular detection of *Leucocytozoon* lineages in São Paulo State. The haemosporidian lineages pDENPET03, hELAALB01, and lELAALB05 were most frequently found, with the first two considered as generalist lineages, as they were previously reported in bird species belonging to different families and, sometimes, even orders [24,25], however, lELAALB05 has been described only in Argentina [26]. In fact, many lineages from this study had been previously detected only in Argentina (pCURCUR01, lDIUDIU11, lELAALB02, hELAALB05, and hELAALB01). Concerning, *Haemoproteus* sp. hELAALB01, it is important to mention that this lineage from Fecchio et al. [27] (GenBank #MK695429 and #MK695430, hELAALB01) is different (96% of identity) from [28] (GenBank #MK981643, #MK264397, #JX029900, hELALB01). The hELALB01 lineage is currently named hMYISWA01 according to MalAvi database. 

Not all infections detected by PCR were confirmed by microscopy. We detected one *Turdus rufiventris* with pTUMIG03 lineage, which has been associated with *P. unalis* [29]. However, although positive for *Plasmodium* sp., characteristics of this species were not observed during microscopic examination of blood smears. Additionally, a smear that was positive for *Haemoproteus* sp. was PCR positive only for *Plasmodium* (pSPMAG06 #HM031936, [30], currently identified as *Plasmodium lutzi* [31]. It is widely known that in samples with co-infections, probably like the latter, general PCR protocols tend to favor the amplification of the parasite with the higher parasitemia or the amplification of the lineage that best matches primer sequences, and mixed infections frequently are overlooked in PCR-based studies [32,33]. 

Here, the presence of gametocytes of *P. nucleophilum* pDENPET03 was documented in *Elaenia albiceps* (Tyrannidae) and *Zonotrichia capensis* (Emberizidae). These findings imply that this parasite has the capacity to complete its life cycle and produce infective gametocytes in the mentioned host species. *Elaenia albiceps* is an endemic bird in the Neotropical region, migrating north between February and March, transiting the Atlantic coast to the Amazon, spending the winter in northeast and northern of Brazil [5,34]. The individuals sampled here were found in the Serra do Mar mountainous forest region in March of each year, during the migration of this species between its breeding areas in southern South America and its winter areas in northeastern Brazil [35,36]. During migration, the birds stay for a few days in the Curucutu region, where they feed on fruits high in the mountainous forest (F. Schunck pers. obs.). 

*Leucocytozoon* lineages from *E. albiceps* showed total similarity with lineages previously described in Argentina, indicating a possible flow of these parasites between this country and Brazil. We do not have evidence of this species being competent hosts (presence of gametocytes in blood smears), but we know that *E. albiceps* from Argentina first fly to the Atlantic coast of Brazil, then to the Cerrado region of central Brazil and no birds overwinter to the west of the Andes mountain range (i.e., Peru, Ecuador and Colombia) [34]. We also found lTROAED02 in *E. albiceps,* a lineage initially identified in Peru in *Troglodytes aedon* [37], but quite generalist in Colombia, where it can infect 25 different species [38]. 

Fecchio et al. [39] linked the scarcity of *Leucocytozoon* infections with warmer temperatures in the tropical lowlands rather than a lack of transmission opportunities, as the vectors for this genus (black flies in the family Simuliidae) are abundant and diverse in lowland regions. However, the warm temperature conditions in the tropical lowlands hardly are limited factors for this infection transmission because *Leucocytozoon* parasites are prevalent in similarly warm tropical rainforests in Africa and Asia [40]. The dissimilarity of vector species could explain the lack of transmission in the study area. We hypothesize that *Leucocytozoon* spp. infect *E. albiceps* in Patagonia (where is cold and there is greater abundance of black flies) and the *Leucocytozoon* transmission ends when the birds enter Brazil. In fact, *Leucocytozoon* sp. presents an inverse latitudinal gradient in the probability of infection and phylogenetic diversity in New World birds, with higher prevalence and lineage diversity toward the poles [26]. The greater probability of a bird becoming infected with *Leucocytozoon* sp. in regions with colder summers and towards the poles, such as the Patagonia region of Argentina, which has a higher prevalence of these parasites than Brazil, indicates that it is possible that *E. albiceps* may be transporting these pathogens during their migratory trips. However, our failure to detect these parasites in blood smears may indicate that *Leucocytozoon* sp. did not evolve to complete its life cycle and produce gametocytes in this host species due to possible abortive development in non-adapted hosts [41]. In this case, only tissue stages develop, and their merozoites or remnants of tissue stages (syncytia) appear in circulation providing templates for PCR amplification, but parasite cannot inhabit red blood cells and thus are difficult to detect by microscopic examination of blood films [24,42]. The birds of genus *Elaenia* would then be a dead end host for the parasite, as it would be unable to infect vector species. It is possible, that parasites may persist in tissue stages during migration and gametocytes are absent, but a relapse may occur at breeding sites and gametocytes would re-appear. In fact, *Leucocytozoon* sp. has not yet been reported in blood smears by microscopic examination in Brazilian birds, potentially missing infections in migratory species where gametocytes were absent. Although, possible not the case for *Leucocytozoon* parasites, our work suggests that *E. albiceps* does indeed have the potential to disperse haemosporidians over long distances, but that such dispersion may be taxonomically restricted to certain genera and lineages, probably generalist lineages such as pDENPET03.

## 4. Materials and Methods 

### 4.1. Sampling

**Figure 7:**
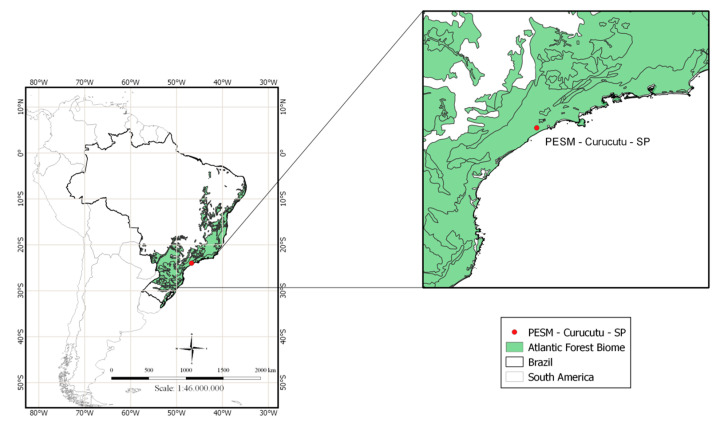
The Núcleo Curucutu sampling site located inside the Atlantic Forest biome, Brazil. The red dot is a central geographic coordinate.

This study was performed in the Núcleo Curucutu, Parque Estadual Serra do Mar (PESM) (23°85′60″ S, 46°83′90″ W, 800 m a.s.l.), in an Atlantic Forest remnant (Figure 7). All birds were caught with mist nets between 2016 and 2019. From each individual, approximately 10 μL of blood was collected from the brachial vein and stored on Whatman^®^ FTA^®^ cards (Whatman, Sigma-Aldrich, Darmstadt, Germany). For 135 bird samples collected in 2019, one or two thin blood smears were also prepared. All blood samples and birds were collected and handled under appropriate permits in Brazil. The project was approved by the Ethics in Use Committee of Animals -CEUA of the Institute of Tropical Medicine - USP (Approval number 2019/000412A and date of approval 08/16/2019).

### 4.2. Microscopic Examination

Thin blood smears were fixed with 100% methanol on the same day of collection and stained with a 10% Giemsa solution, within 30 days after collections, for 1 h [12]. Blood smears were then examined microscopically for 20–25 min by viewing 100 fields at low magnification (400×) and 100 fields at high magnification (1000×) [12], using a Leica^®^ DM3000LED light microscope. Morphological identification of parasite species was performed according to Valkiūnas [12] and Valkiūnas and Iezhova [43]. 

### 4.3. Molecular Detection and Genotyping of Haemosporidian Infections

DNA from blood samples was extracted with the Wizard^®^ SV 96 Genomic DNA Purification System (Promega, Madison, WI, USA) with modifications. Briefly, FTA cards with 10 μL of blood were incubated with Whole Blood Lysis Buffer (400 μL) for 15 min in a shaker at 90 °C. The initial lysis was completed with Proteinase K and incubated overnight in a shaker at 37 °C. The lysates were transferred to columns and washed according to the manufacturer’s instructions. DNA was eluted in 50 μL of Nuclease-FreeWater and stored at −20 °C.

Polymerase chain reactions (PCR) were conducted using a nested protocol targeting the mitochondrial cytochrome *b* (*cytb*) gene of *Plasmodium*, *Haemoproteus*, and *Leucocytozoon* species [44]. The first reaction used the primers HaemNFI/HaemNR3 and 50 ng of genomic DNA. In the nested reaction, performed with a second pair of primers (HaemF/HaemR2 for *Plasmodium* and *Haemoproteus* or HaemFL/HaemR3L for Leucocytozoon), 1 µL of the product from the first reaction was used as a template. In each PCR, positive controls were carried out in parallel, containing *Plasmodium*, *Haemoproteus*, and *Leucocytozoon* DNA, and ultrapure water served as a negative control. 

PCR products were sequenced by BigDye^®^ Terminator v3.1 Cycle Sequencing Kit in ABI PRISM^®^ 3500 Genetic Analyzer (Applied Biosystems, Carlsbad, CA, USA), using nested PCR primers. The *cytb* sequences (~480 bp) were obtained and aligned with sequences from the MalAvi database (http://130.235.244.92/Malavi/), in order to verify parasite lineage identity and identify new lineages. The sequences possessing at least one different nucleotide were considered unique lineages and were named according to the MalAvi nomenclature [45] and deposited in GenBank and MalAvi database.

A comparison between the prevalence of haemosporidian infections (detected by PCR) between resident birds and migratory birds was analysed using a chi-square test with Yates’ correction for smaller samples as warranted. Findings were considered statistically significant if *p* < 0.05. 

### 4.4. Phylogenetic Analysis

The phylogenetic relationship among reported parasites was inferred using partial *cytb* gene sequences. GenBank accessions of the used sequences are given in the phylogenetic trees. The phylogenetic reconstruction was performed separately for *Plasmodium*, *Haemoproteus*, and *Leucocytozoon* parasites using the Bayesian inference method implemented in MrBayes v3.2.0 [46]. Bayesian inference was executed with two Markov Chain Monte Carlo searches of 3 million generations, with each sampling 1 of 300 trees. After a burn-in of 25%, the remaining 15,002 trees were used to calculate the 50% majority-rule consensus tree. The phylogeny was visualized using FigTree version 1.4.0 [47].

## Figures and Tables

**Table 1:** Morphometric data of host cells and mature gametocytes of *Haemoproteus nucleocentralis* n. sp. (cytochrome *b* lineage hTANDES01).

Feature		Measurements ^a^
Uninfected erythrocyte	Length	11.1–13.3 (12.0 ± 0.7)
	Width	6.5–7.7 (7.0 ± 0.4)
	Area	58.6–80.2 (66.4 ± 5.3)
Uninfected erythrocyte nucleus	Length	5.1–6.4 (5.8 ± 0.3)
	Width	2.7–3.4 (3.1 ± 0.2)
	Area	13.5–17.9 (15.1 ± 1.2)
Macrogametocyte		
Infected erythrocyte	Length	11.7–13.7 (12.5 ± 0.5)
	Width	5.8–8.1 (6.9 ± 0.6)
	Area	60.8–85.8 (70.4 ± 6.8)
Infected erythrocyte nucleus	Length	4.3–6.3 (5.1 ± 0.4)
	Width	2.3–3.9 (3.2 ± 0.4)
	Area	9.4–18.9 (13.3 ± 1.8)
Gametocyte	Length	13.0–17.7 (14.8 ± 1.2)
	Width	1.5–3.6 (2.4 ± 0.6)
	Area	31.7–47.5 (39.5 ± 4.8)
Gametocyte nucleus	Length	2.2–4.3 (2.8 ± 0.5)
	Width	1.1–3.0 (1.8 ± 0.6)
	Area	2.1–6.5 (3.9 ± 1.3)
Pigment granules		7.0–12.0 (10.1 ± 1.6)
NDR ^c^		0.2–1.1 (0.7 ± 0.2)
Microgametocyte (*n* = 17)		
Infected erythrocyte	Length	11.5–14.5 (12.7 ± 0.8)
	Width	6.2–8.2 (7.5 ± 0.5)
	Area	67.0–84.0 (73.9 ± 4.6)
Infected erythrocyte nucleus	Length	4.8–5.9 (5.3 ± 0.4)
	Width	2.5–3.9 (3.0 ± 0.4)
	Area	11.1–15.2 (13.1 ± 1.1)
Gametocyte	Length	11.9–17.3 (14.5 ± 1.3)
	Width	2.2–3.7 (3.0 ± 0.4)
	Area	34.1–49.9 (43.1 ± 5.0)
Gametocyte nucleus ^b^	Length	-
	Width	-
	Area	-
Pigment granules		7.0–17.0 (10.6 ± 2.7)
NDR ^c^		0.4–0.9 (0.7 ± 0.2)

^a^ Number of measurements (*n*) was 21, except indicated otherwise. All measurements are given in micrometres, except for pigment granules. Minimum and maximum values are provided, followed in parentheses by the arithmetic mean and standard deviation. ^b^ Microgametocyte nuclei were hardly defined and difficult to measure. ^c^ Nucleus displacement ratio (NDR) according to [16].

**Table 2:** Numbers of samples from resident bird species sampled in Núcleo Curucutu, Parque Estadual Serra do Mar, SP, Brazil; numbers in parentheses represent the samples positive for haemosporidian parasites.

Order Family	Host Species	No. Sampled (No. Positive)	Parasite and Lineages	GenBank Accession
**Apodiformes**				
Trochilidae	*Amazilia fimbriata*	1		
	*Aphantochroa cirrochloris*	1		
	*Heliodoxa rubricauda*	2 (1)	*Plasmodium* sp.	
			pVIOLI03	MW394211
	*Leucochloris albicollis*	1		
	*Phaethornis eurynome*	1		
	*Ramphodon naevius*	15		
	*Thalurania glaucopis*	2		
**Caprimulgiformes**				
Caprimulgidae	*Hydropsalis forcipata*	1		
**Columbiformes**				
Columbidae	*Geotrygon montana*	2		
**Galbuliformes**				
Bucconidae	*Malacoptila striata*	1		
**Passeriformes**				
Coerebidae	*Coereba flaveola*	2		
Conopophagidae	*Conopophaga lineata*	4 (2)	*Plasmodium* sp. pCONLIN16	MT724554
			*Plasmodium* sp. pLEAMAN01	MT724398
Cotingidae	*Schiffornis virescens*	3		
Dendrocolaptidae	*Dendrocincla turdina*	1		
	*Sittasomus griseicapillus*	1		
	*Xiphorhynchus fuscus*	3		
Emberizidae	*Zonotrichia capensis*	6 (3)	*P. nucleophilum* pDENPET03	MT724565
			*H. erythrogravidus* hZOCAP01	MT724564 MT724567
Furnariidae	*Heliobletus contaminatus*	6		
	*Lochmias nematura*	2 (1)	*Plasmodium* sp. pTURFAL01	MT724472
	*Synallaxis spixi*	2		
Parulidae	*Basileuterus culicivorus*	15 (1)	*Plasmodium* sp. pBASCUL01	MT724400
	*Basileuterus leucoblepharus*	2		
	*Geothlypis aequinoctialis*	17 (1)	*Plasmodium* sp. pGEOTRI01	MT724529
	*Parula pitiayumi*	3		
Pipridae	*Chiroxiphia caudata*	2		
Platyrinchidae	*Platyrinchus mystaceus*	1		
Thamnophilidae	*Batara cinerea*	2		
	*Drymophila malura*	3		
	*Dysithamnus mentalis*	1 (1)	*Plasmodium* sp. pPYLEU01	MT724470
	*Thamnophilus caerulescens*	6		
Thraupidae	*Hemithraupis ruficapilla*	1		
	*Stephanophorus* *diadematus*	6		
	*Tachyphonus coronatus*	8 (2)	*Plasmodium* sp. pTARUF01	MT724541
			*Plasmodium* sp. pLEPCOR05	MT724536
	*Tangara desmaresti*	1 (1)	*H. nucleocentralis* hTANDES01	MT724553
	*Trichothraupis melanops*	5		
Troglodytidae	*Troglodytes aedon*	1 (1)	*P. nucleophilum* pDENPET03	MT724566
Turdidae	*Turdus leucomelas*	1 (1)	*P. lutzi* pSPMAG06	MT724556
	*Turdus rufiventris*	2 (1)	*Plasmodium* sp. pTUMIG03	MT724559
Tyrannidae	*Attila rufus*	1		
	*Camptostoma obsoletum*	2		
	*Cyclarhis gujanensis*	2		
	*Hemitriccus diops*	1		
	*Hemitriccus nidipendulus*	5		
	*Hylophilus poicilotis*	7		
	*Leptopogon* *amaurocephalus*	1		
	*Mionectes rufiventris*	8		
	*Myiobius atricaudus*	1		
	*Poecilotriccus plumbeiceps*	4		
	*Phylloscartes ventralis*	5		
**Piciformes**				
Picidae	*Picumnus temminckii*	3		
	*Veniliornis spilogaster*	2		
**Psitaciformes**				
Psittacidae	*Pyrrhura frontalis*	5		
**Total Residents**		**181 (16)**		

**Table 3:** Numbers of samples from migratory bird species sampled in Núcleo Curucutu, Parque Estadual Serra do Mar, SP, Brazil; numbers in parentheses represent the samples positive for haemosporidian parasites. Samples from *Elaenia albiceps* are highlighted in gray.

Order Family	Host Species	No. Sampled (No. Positive)	Parasites and Lineages	GenBank Accession
**Passeriformes**				
Emberizidae	*Haplospiza unicolor*	11 (1)	*Plasmodium* sp. pRAMCAR05	MT724551
Thraupidae	*Pipraeidea melanonota*	1		
Turdidae	*Turdus amaurochalinus*	1		
	*Turdus flavipes*	3 (1)	*P. nucleophilum* pDENPET03	MT724555
Tyrannidae	*Attila phoenicurus*	1		
	*Elaenia albiceps*	150 (37)	*P. homocircumflexum* pCOLL4	MT724561
			*Plasmodium* sp. pELAALB07	MT724471
			*P. nucleophilum* pDENPET03	MT724542/ MT724547/ MT724545
			*Plasmodium* sp. pPADOM09	MT724539/ MT724533/MT724397
			*Plasmodium* sp. pPADOM11	MT724550
			*Plasmodium* sp. pPHPAT01	MT724562
			*Plasmodium* sp. pLEPCOR05	MT724546
			*Haemoproteus* sp. hMYISWA01	MT724399
			*Haemoproteus* sp. hELAALB01	MT724538/ MT724543/ MT724549/ MT724557/ MT724537/ MT724544/ MT724548/ MT724560
			*Leucocytozoon* sp. lDIUDIU11	MW394193-MW394196
			*Leucocytozoon* sp. lELAALB02	MW394197-MW394200
			*Leucocytozoon* sp. lELAALB05	MW394201-MW394207
			*Leucocytozoon* sp. lTROAED02	MW394208-MW394209
			*Leucocytozoon* sp. lZOLPYR01	MW394210
	*Elaenia mesoleuca*	13 (1)	*Plasmodium* sp. pTRMEL02	MT724468
	*Empidonomus varius*	1		
	*Knipolegus cyanirostris*	7		
	*Lathrotriccus euleri*	1		
	*Myiarchus swainsoni*	7 (1)	*Haemoproteus* sp. hMYISWA01	MT724530
Tyrannidae	*Myiodynastes maculatus*	3		
	*Tyrannus melancholicus*	2 (1)	*Haemoproteus* sp. hMYISWA01	MT724535
Vireonidae	*Vireo olivaceus*	17 (10)	*P. nucleophilum* pDENPET03	MT724563/ MT724540
			*Plasmodium* sp. pVIOLI03	MT724527/ MT724531/ MT724532/MT724534/ MT724552
			*Haemoproteus* sp. hVIGIL09	MT724469
			*Haemoproteus* sp. hCHIPAR01	MT724528
			*Haemoproteus* sp. hVIOLI05	MT724558
**Total Migratory**		**218 (52)**		

Two migratory taxa refer to the subspecies *Elaenia albiceps chilensis* and *Vireo olivaceus chivi*, recognized as species by the Brazilian Committee for Ornithological Records [1].

## Data Availability

The data presented in this study are available in Supplementary Material.

## Supplementary Materials

The following are available online at https://www.mdpi.com/2076-0817/10/2/103/s1, Supplementary Table S1: Haemosporidian parasite lineages identified in this study, and records on MalAvi Database of their occurrence and transmission locations. M, migratory; R, resident. Supplementary Raw Data Spreadsheet S2.
