# Supplementary material for: Avian Malaria and Related Parasites from Resident and Migratory Birds in the Brazilian Atlantic Forest, with Description of a New Haemoproteus Species
Source: Pathogens. 2021 Jan 21;10(2):103. doi: 10.3390/pathogens10020103 (PMC7912314; doi:10.3390/pathogens10020103)
Supplement: Supplementary file 1 [file pathogens-10-00103-s001.zip › SupplementaryTable S1 xml.pdf]

pathogens MDPI

Supplementary Information

Table 1. Haemosporidian parasite lineages identified in this study, and records of their occurrence on MalAvi Database. M, migratory; R, resident.

This Study		Malavi Database		
**Lineage (Genbank n°)**	**Host**	**Hosts**	**Country**	**Reference Name**
pBASCUL01 (MT724400)	***R: Basileuterus culicivorus***			
pCOLL4 (DQ368374)	*M: Elaenia albiceps*	*Carduelis spinus, Lanius collurio, Loxia curvirostra, Sturnus vulgaris*	Russia	[1], [2]
		Coryphistera alaudina, Gnorimopsar chopi, Limnornis curvirostris, Mimus saturninus, Poospiza lateralis, Stephanophorus diadematus	Uruguay	[3]
		Ficedula albicollis	Sweden<br>Hungary	[4], [5]<br>[6],
		Cyclarhis gujanensis<br>*Dolichonyx oryzivorus*<br>*Foudia omissa, Hartlaubius auratus*<br>*Pycnonotus barbatus*	Brazil<br>United States<br>Madagascar<br>Benin	[7]<br>[8]<br>[9]<br>[10]
pCONLIN16 (JX021452)	*R: Conopophaga lineata*	*Basileuterus flaveolus*, ***Conopophaga lineata***	Brazil	[11]
pCURCUR01 *P. homopolare* (pTURFAL01) (MT724472)	*R: Lochmias nematura*	***Curaeus curaeus, Turdus falcklandii***	Argentina	[7]
pDENPET03 (KU562464)<br>*P. nucleophilum*	*M: Elaenia albiceps*<br>*R: Troglodytes aedon*<br>*M: Turdus flavipes*<br>*M: Vireo olivaceus*<br>*R: Zonotrichia capensis*	*Alopochen aegyptiacus, Ammodramus humeralis, Arremon taciturnus, Basileuterus flaveolus, Basileuterus leucoblepharus, Cacicus solitarius, Cantorchilus longirostris, Coereba flaveola, Colaptes melanochloros, Coryphistera alaudina, Coryphospingus pileatus, Cyclarhis gujanensis, Cygnus atratus, Cypsnagra hirundinacea, Formicivora melanogaster, Guaruba guarouba, Gubernatrix cristata, Hemithraupis guira, Mimus saturninus, Myiarchus swainsoni, Myiothlypis flaveola, Neothraupis fasciata, Netta erythrophthalma, Pachyramphus polychopterus, Parula pitiayumi, Passer*	Brazil	[7], [11], [12], [13], [14], [15], [16], [17]; [18], [19]

		domesticus, Phoenicopterus chilensis, Pipile jacutinga, Psarocolius decumanus, Ramphocelus carbo, Ramphastos toco, Ramphastos vitellinus, Rynchops niger, Saltator coerulescens, Schoeniophylax phryganophilus, Spheniscus magellanicus, Synallaxis frontalis, Thamnophilus nigrocinereus, Thryothorus longirostris, Trichothraupis melanops, Turdus albicolis, Turdus leucomelas, Turdus rufiventris, Volatinia jacarina, Zonotrichia capensis		
		Anas discors, ***Dendroica petechia,*** *Dolichonyx oryzivorus, Dumetella carolinensis, Geothlypis trichas, Petrochelidon pyrrhonota, Riparia riparia, Setophaga petechia, Turdus migratorius, Vireo griseus*	United States	[8], [20], [21], [22], [23], [24], [25]
		Basileuterus culicivorus, Basileuterus leucoblepharus, Cranioleuca pyrrhophia, Gnorimopsar chopi, Zonotrichia capensis	Uruguay	[3]
		Automolus rufipileatus, Arremon taciturnus, Cyphorhinus arada, Hypocnemis subflava, Pipra fasciicauda, Ramphocelus carbo, Turdus hauxwelli, Xiphorhynchus ocellatus	Peru	[16]
		Carduelis barbata<br>*Dendroica coronata*	Argentina<br>Canada	[7]<br>[26]
		Cacicus cela, Cacicus haemorrhous, Diopsittaca nobilis, Volatinia jacarina	Guyana	[3]
pELAALB07 (MT724471)	***M: Elaenia albiceps***			
pGEOTRI01 (MF817777)	*R: Geothlypis aequinoctialis*	*Catharus ustulatus, Dendroica magnólia, Dendroica palmarum,* ***Geothlypis trichas,*** *Helmitheros vermivorum, Melospiza lincolnii*	United States	[20], [23], [25], [27]
		Atlapetes albinucha<br>*Euneornis campestris*<br>*Melospiza melodia. Zonotrichia albicollis*<br>*Seiurus noveboracensis*	Colombia<br>Unknown<br>Canada<br>Venezuela	[28]<br>[29]<br>[30], [31]<br>[23], [32], [33],
pLEAMA01 (JX021454)	*R: Conopophaga lineata*	***Leptopogon amaurocephalus***<br>*Conopophaga lineata*	Brazil	[11]

pLEPCOR05 (pLECOR04) (KU236434) (pVOLJAC03)	*R: Tachyphonus coronatus*<br>*M: Elaenia albiceps*	*Coryphospingus pileatus*, ***Lepidothrix coronata,*** *Volatinia jacarina*<br>*Tangara schrankii*	Brazil<br>Peru	[7], [17], [34], [35]<br>[7]
pPADOM09 (AF069611)<br>*P. elongatum*	*M: Elaenia albiceps*	*Ammodramus humeralis, Anser cygnoides, Asthenes pyrrholeuca, Basileuterus culicivorus, Carduelis barbata, Cnemotriccus fuscatus, Coryphospingus cucullatus, Coryphospingus pileatus, Dendrocolaptes certhia, Donacobius atricapilla, Elaenia albiceps, Elaenia cristata, Elaenia spectabilis, Furnarius leucopus, Haplospiza unicolor, Lathrotriccus euleri Myiarchus swainsoni, Myiarchus tyrannulus, Myiopagis viridicata, Myiophobus fasciatus, Neothraupis fasciata, Paroaria capitata, Passer domesticus, Pheugopedius genibarbis, Pitangus sulphuratus, Puffinus puffinus, Ramphotrigon ruficauda, Rhytipterna simplex, Saltator coerulescens, Spheniscus magellanicus, Synallaxis scutata, Tachyphonus coronatus, Tachyphonus cristatus, Tachyphonus phoenicius, Thraupis palmarum, Thryothorus genibarbis, Trichothraupis melanops, Troglodytes aedon, Troglodytes musculus, Tyrannus melancholicus, Zonotrichia capensis*	Brazil	[7], [11], [12], [13], [15], [16], [17], [18], [36]
		Dendroica coronata, Dolichonyx oryzivorus, Geothlypis trichas, Larosterna inca, Mniotilta varia, Passer domesticus, Tachycineta thalassina	United States	[8], [22], [27], [37], [38]
		Gnorimopsar chopi, Pseudoleistes guirahuro, Stephanophorus diadematus, Tangara preciosa, Troglodytes aedon, Turdus rufiventris	Uruguay	[3], [37]
		Basileuterus culicivorus, Coryphospingus cucullatus, Elaenia albiceps, Elaenia mesoleuca, Troglodytes aedon, Zonotrichia capensis	Argentina	[7], [39]
		Elaenia albiceps, Troglodytes musculus	Chile	[40]
		Campylorhynchus yucatanicus<br>*Troglodytes aedon*	Unknown<br>Peru	[29]<br>[41]
pPADOM11 (HM146899)	*M: Elaenia albiceps*	*Aegolius acadicus, Agelaius phoeniceus, Baeolophus bicolor, Cardinalis cardinalis, Carduelis*	United States	[8], [12], [25], [27], [38], [42], [43], [44], [45], [46], [47],

		tristes, Carpodacus mexicanus, Colaptes auratus, Dolichonyx oryzivorus, Dumetella carolinensis, Gavia immer, Quiscalus quiscula, Melospiza melodia, ***Passer domesticus,*** *Passerina cyanea, Pheucticus ludovicianus, Sialia sialis, Somateria spp, Spheniscus demersus, Spizella passerina, Spizella pusilla, Strix varia, Turdus migratorius*		
		Basileuterus flaveolus, Camptostoma obsoletum, Campylorhamphus trochilirostris, Coereba flaveola, Coryphospingus pileatus, Dacnis cayana, Neothraupis fasciata, Passer domesticus, Polioptila plúmbea, Saltator similis, Sittasomus griseicapillus, Tachyphonus phoenicius, Tachyphonus rufus, Thlypopsis sordida, Trichothraupis melanops, Volatinia jacarina	Brazil	[7], [11], [12], [16], [17],
		Cacicus cela, Cyanocompsa cyanoides, Saltator grossus, Saltator maximus	Guyana	[3]
		Melospiza melodia, Tachycineta bicolor	Canada	[30], [49]
		Catharus minimus<br>*Chrysomus ruficapillus, Polioptila dumicola*<br>*Mniotilta varia, Turdus fumigatus, Vireo griseus*<br>*Volatinia jacarina*	Colombia<br>Uruguay<br>Unknown<br>Peru	[49]<br>[3]<br>[29]<br>[16]
pPYLEU01 (JX021484)	*R: Dysithamnus mentalis*	*Dysithamnus plumbeus,* ***Pyriglena leucoptera***	Brazil	[11]
pRAMCAR05 (KU562679)	*M: Haplospiza unicolor*	***Ramphocelus carbo***	Brazil	[16]
pSPMAG06 (HM031936)<br>*P. lutzi*	*R: Turdus leucomelas*	*Musophaga violacea,* ***Spheniscus magellanicus***<br>*Turdus rufiventris*	Brazil	[13], [50], [51],<br>[7], [52]
pTARUF01 (JX021475)	*R: Tachyphonus coronatus*	*Basileuterus flaveolus, Saltator similis,* ***Tachyphonus rufus,*** *Tangara cayana, Thraupis palmarum*	Brazil	[7], [11],
pTRMEL02 (JX021468)	*M: Elaenia mesoleuca*	*Zonotrichia capensis*	Argentina	[39]
		Anabazenops fuscus, ***Trichothraupis melanops,*** *Vireo olivaceus, Zonotrichia capensis*	Brazil	[11]
pTUMIG03 (KU562788)	*R: Turdus rufiventris*	*Catharus ustulatus, Empidonax hammondii, Hylocichla mustelina, Icterus galbula, Larosterna inca,*	United States	[20], [23], [24], [25], [27], [38], [45], [54],

		Regulus calêndula, Sturnus vulgaris, Toxostoma rufum, ***Turdus migratorius,*** *Vermivora celata*		
		Spheniscus magellanicus, Turdus albicollis, Turdus amaurochalinus, Turdus flavipes, Turdus subalaris, Turdus rufiventris	Brazil	[7], [11], [15], [16],
		Entomodestes leucotis, Troglodytes aedon, Turdus nigriceps, Turdus serranus	Peru	[7], [41]
		Myioborus miniatus, Turdus albicollis, Turdus flavipes, Turdus fuscater, Turdus grayi, Turdus olivater	Colombia	[49]
		Anairetes fernandezianus<br>*Turdus falcklandii, Zonotrichia capensis*<br>*Turdus rufiventris*	Chile<br>Argentina<br>Uruguay	[55]<br>[40], [55]<br>[3], [51]
pVIOLI03 (JX029897)	*R: Heliodoxa rubricauda*<br>*M: Vireo olivaceus*	*Troglodytes aedon,* ***Vireo olivaceus***<br>***Vireo olivaceus***<br>***Vireo olivaceus***	Brazil<br>United States<br>Peru	[11]<br>[25]<br>[7]
pPHPAT01 (KY305005)	*M: Elaenia albiceps*	*Ammodramus humeralis, Basileuterus flaveolus Basileuterus leucoblepharus, Coryphospingus pileatus, Paroaria dominicana, Phaeomyias murina, Rynchops niger, Spheniscus magellanicus, Tangara cyanoptera*	Brazil	[7], [11], [14], [15], [17], [52]
		Bubo virginianus, Cardinalis cardinalis, Dolichonyx oryzivorus, Geothlypis trichas, Larosterna inca, Quiscalus quiscula	United States	[8], [22], [25], [38], [44]
		Phrygilus patagonicus<br>*Sayornis nigricans*	Chile<br>Peru	[40]<br>[53]
hELAALB01 (MK695430)	*M: Elaenia albiceps*	*Anairetes parulus,* ***Elaenia albiceps***	Argentina	[7]
hMYISWA01 (KU562174)	*M: Elaenia albiceps*<br>*M: Myiarchus swainsoni*<br>*M: Tyrannus melancholicus*	*Elaenia chiriquensis,* ***Myiarchus swainsoni*** *Myiodynastes maculatus, Myiopagis viridicata, Phaeomyias murina, Phaethornis malaris, Serpophaga subcristata*	Brazil	[7], [16]
hTANDES01 (MT724553)	***R: Tangara desmaresti***			
hVIGIL09 (hCOLPLU01) (MH457345)	*M: Vireo olivaceus*	*Euphonia xanthogaster, Microcerculus marginatus Myiodynastes maculatus, Myiornis albiventris*	Peru	[7]

		Patagioenas plumbea, Turdus nigriceps, ***Vireo gilvus,*** *Willisornis poecilinotus*		
		Vireo gilvus<br>*Vireo olivaceus*	United States<br>Brazil	[25]<br>[7]
hVIOLI05 (hVIREO02) (KF482350)	*M: Vireo olivaceus*	***Vireo olivaceus***	Peru<br>Colombia<br>Brazil	[53]<br>[28]<br>[7]
hCHIPAR01 (hVIROLI05) (MT724528)	*M: Vireo olivaceus*	***Chiroxiphia pareola***	Peru	[41]
hZOCAP01 (EF153649)	*R: Zonotrichia capensis*	***Zonotrichia capensis***	Chile<br>Peru<br>Colombia<br>Brazil Argentina<br>Ecuador<br>Costa Rica	[39], [40],<br>[39], [53], [56]<br>[28], [39], [57],<br>[7]<br>[7]<br>[39], [57],<br>[39]
lDIUDIU11 (MK947686)	*M: Elaenia albiceps*	***Diuca diuca***<br>*Aphrastura spinicauda*	Argentina<br>Chile	[7]<br>[58]
lELAALB02 (MK947541)	*M: Elaenia albiceps*	***Elaenia albiceps***<br>*Aphrastura spinicauda*	Argentina<br>Chile	[7]<br>[58]
lELAALB05 (MK947689)	*M: Elaenia albiceps*	***Elaenia albiceps***<br>*Aphrastura spinicauda*	Argentina<br>Chile	[7]<br>[58]
lZOLPYR01 (MK947545)	*M: Elaenia albiceps*	***Xolmis pyrope***<br>*Aphrastura spinicauda*	Argentina<br>Chile	[7]<br>[58]
lTROAED02 (KF767431)	*M: Elaenia albiceps*	***Troglodytes aedon***<br>*Anabacerthia striaticollis, Arremon schlegeli, Atlapetes melanocephalus, Basileuterus basilicus, Basileuterus conspicillatus, Buarremon torquatus, Catamenia homochroa, Catharus fuscater, Cranioleuca hellmayri, Diglossa albilatera, Elaenia frantzii, Henicorhina anachoreta, Henicorhina leucophrys, Mecocerculus leucophrys, Mionectes olivaceus, Myiarchus tuberculifer, Myioborus flavivertex, Myioborus miniatus, Myrmotherula schisticolor, Pipra erythrocephala, Pyrrhomyias cinnamomeus, Turdus albicollis, Turdus flavipes, Turdus fuscater, Zonotrichia capensis*	Peru<br>Colombia	[41]<br>[49]

References:

1. Palinauskas, V.; Žiegytė, R.; Ilgūnas, M.; Iezhova, T.A.; Bernotienė, R.; Bolshakov, C.; Valkiūnas, G. Description of the first cryptic avian malaria parasite, *Plasmodium homocircumflexum* n. sp., with experimental data on its virulence and development in avian hosts and mosquitoes. *Int. J. Parasitol.* **2015**, *45*, 51–62. [https://doi.org/10.1016/j.ijpara.2014.08.012] [https://pubmed.ncbi.nlm.nih.gov/25449950/]
2. Ilgūnas, M.; Bukauskaitė, D.; Palinauskas, V. *et al.* Mortality and pathology in birds due to *Plasmodium (Giovannolaia) homocircumflexum* infection, with emphasis on the exoerythrocytic development of avian malaria parasites. *Malar. J.* **2016**, *15*, 256 [https://doi.org/10.1186/s12936-016-1310-x] [http://www.ncbi.nlm.nih.gov/pmc/articles/pmc4857288/]
3. Durrant, K.; Beadell, J.; Ishtiaq, F.; Graves, G.; Olson, S.; Gering, E.; . . . Fleischer, R. Avian Hematozoa in South America: A Comparison of Temperate and Tropical Zones. *Ornithol. Monographs.* **2006**, *60*, 98-111. [https://doi.org/10.2307/40166831]
4. Kulma, K.; Low, M.; Bensch, S.; Qvarnström, A. Malaria infections reinforce competitive asymmetry between two Ficedula flycatchers in a recent contact zone. *Mol Ecol.* **2013**, *22*, 4591–4601. [https://doi.org/10.1111/mec.12409] [https://pubmed.ncbi.nlm.nih.gov/23980765/]
5. Kulma, K.; Low, M.; Bensch, S.; Qvarnström, A. Malaria-infected female collared flycatchers (*Ficedula albicollis*) do not pay the cost of late breeding. *PLoS One*. **2014**, *9*, e85822. [https://doi.org/10.1371/journal.pone.0085822] [http://www.ncbi.nlm.nih.gov/pmc/articles/pmc3900437/]
6. Garamszegi, L. Z.; Zagalska-Neubauer, M.; Canal, D.; Markó, G.; Szász, E.; Zsebők, S.; Szöllősi, E.; Herczeg G.; Török, J.; Malaria parasites, immune challenge, MHC variability, and predator avoidance in a passerine bird, *Behav. Ecol.* **2015**, *26*, 1292–1302. [https://doi.org/10.1093/beheco/arv077]
7. Fecchio, A., Bell, J.A.; Pinheiro, R.B.P.; Cueto, V.R.; Gorosito, C.A.; Lutz, H;L; Gaiotti, M.G.; Paiva, L.V.; França, L.F.; Toledo-Lima, G.; Tolentino, M.; Pinho, J.B.; Tkach, V.V.; Fontana, C.S.; Grande, J.M.; Santillán, M.A.; Caparroz, R.; Roos, A.L.; Bessa, R.; Nogueira, W.; Moura, T.; Nolasco, E.C.; Comiche, K.J.M.; Kirchgatter, K., Guimarães, L.O.; Dispoto, J.H.; Marini, M.Â.; Weckstein, J.D.; Batalha-Filho, H.; Collins, M.D. Avian host composition, local speciation and dispersal drive the regional assembly of avian malaria parasites in South American birds. *Mol. Ecol.* **2019**, *28*, 2681-2693. [https://doi.org/10.1111/mec.15094]
8. Levin, I. I.; Zwiers, P.; Deem, S. L.; Geest, E. A.; Higashiguchi, J. M.; Iezhova, T. A.; Jiménez-Uzcátegui, G.; Kim, D. H.; Morton, J. P.; Perlut, N. G.; Renfrew, R. B.; Sari, E. H.; Valkiunas, G.; Parker, P. G. Multiple lineages of Avian malaria parasites (*Plasmodium*) in the Galapagos Islands and evidence for arrival via migratory birds. *Conserv. Biol.* **2013**, *27*, 1366–1377. [https://doi.org/10.1111/cobi.12127] [https://pubmed.ncbi.nlm.nih.gov/24033638/]
9. Musa, S.; Mackenstedt, U.; Woog, F.; Dinkel, A. Avian malaria on Madagascar: prevalence, biodiversity and specialization of haemosporidian parasites. *Int. J. Parasitol.* **2019**, *49*, 199–210. [https://doi.org/10.1016/j.ijpara.2018.11.001] [https://pubmed.ncbi.nlm.nih.gov/30471288/]
10. Harvey, J.A.; Voelker, G. Avian haemosporidian detection across source materials: prevalence and genetic diversity. *Parasitol. Res*. **2017**, *116*, 3361–3371 [https://doi.org/10.1007/s00436-017-5654-0]
11. Lacorte, G. A.; Félix, G. M.; Pinheiro, R. R.; Chaves, A. V.; Almeida-Neto, G.; Neves, F. S.; Leite, L. O.; Santos, F. R.; Braga, E. M. Exploring the diversity and distribution of neotropical avian malaria parasites--a molecular survey from Southeast Brazil. *PLoS One.* **2013**, *8*, e57770. [https://doi.org/10.1371/journal.pone.0057770] [http://www.ncbi.nlm.nih.gov/pmc/articles/pmc3585926/]
12. Marzal, A.; Ricklefs, R.E.; Valkiūnas, G.; Albayrak, T.; Arriero, E.; Bonneaud, C.; Czirják, G.A.; Ewen, J.; Hellgren, O.; Hořáková, D.; Iezhova, T.A.; Jensen, H.; Križanauskienė, A.; Lima, M.R.; de Lope, F.; Magnussen, E.; Martin, L. B.; Møller, A. P.; Palinauskas, V.; Pap, P. L.; … Bensch, S. Diversity, loss, and gain of malaria parasites in a globally invasive bird. *PLoS One.* **2011**, *6*, e21905. [https://doi.org/10.1371/journal.pone.0021905] [http://www.ncbi.nlm.nih.gov/pmc/articles/pmc3136938/]
13. Chagas, C.R.; Valkiūnas, G.; de Oliveira Guimarães, L.; Monteiro, E.F.; Guida, F.J.; Simões, R.F.; Rodrigues, P.T.; de Albuquerque Luna, E.J.; Kirchgatter, K. Diversity and distribution of avian malaria and related haemosporidian parasites in captive birds from a Brazilian megalopolis. *Malar. J.* **2017**, *16*, 83. [https://doi.org/10.1186/s12936-017-1729-8] [http://www.ncbi.nlm.nih.gov/pmc/articles/pmc5316177/]

14. Roos, F.L.; Belo, N.O.; Silveira, P.; Braga, E.M. Prevalence and diversity of avian malaria parasites in migratory Black Skimmers (*Rynchops niger*, Laridae, Charadriiformes) from the Brazilian Amazon Basin. *Parasitol. Res*. **2015**, *114*, 3903–3911. [https://doi.org/10.1007/s00436-015-4622-9] [https://pubmed.ncbi.nlm.nih.gov/26193823/]

15. Vanstreels, R.E.; da Silva-Filho, R.P.; Kolesnikovas, C.K.; Bhering, R.C.; Ruoppolo, V.; Epiphanio, S.; Amaku, M.; Ferreira Junior, F.C.; Braga, É.M.; Catão-Dias, J.L. Epidemiology and pathology of avian malaria in penguins undergoing rehabilitation in Brazil. *Vet. Res*. **2015**, *46*, 30. [https://doi.org/10.1186/s13567-015-0160-9] [http://www.ncbi.nlm.nih.gov/pmc/articles/pmc4357068/]

16. Fecchio, A.; Svensson-Coelho, M.; Bell, J.; Ellis, V.A.; Medeiros, M.C.; Trisos, C.H.; Blake, J.G.; Loiselle, B.A.; Tobias, J.A.; Fanti, R. et al. Host associations and turnover of haemosporidian parasites in manakins (Aves: Pipridae). *Parasitology*. **2017**, *144*, 984–993. [https://doi.org/10.1017/S0031182017000208] [https://pubmed.ncbi.nlm.nih.gov/28290270/]

17. Ferreira Junior, F. C.; Rodrigues, R. A.; Ellis, V. A.; Leite, L. O.; Borges, M.; Braga, É. M. Habitat modification and seasonality influence avian haemosporidian parasite distributions in southeastern Brazil. *PloS One*. **2017**, *12*, e0178791. [https://doi.org/10.1371/journal.pone.0178791] [http://www.ncbi.nlm.nih.gov/pmc/articles/pmc5456369/]

18. Fecchio, A.; Pinheiro, R.; Felix, G.; Faria, I.P.; Pinho, J.B.; Lacorte, G.A.; Braga, E.M.; Farias, I.P.; Aleixo, A.; Tkach, V.V.; Collins, M.D.; Bell, J.A.; Weckstein, J.D. Host community similarity and geography shape the diversity and distribution of haemosporidian parasites in Amazonian birds. *Ecography*. **2018**, *41*, 505-515. [https://doi.org/10.1111/ecog.03058]

19. De La Torre, G.; Freitas, F.; Fratoni, R.; Guaraldo, A. de Angeli Dutra, D.; Braga, E.; Manica, L.; Hemoparasites and their relation to body condition and plumage coloration of the White-necked thrush (*Turdus albicollis*). *Ethol. Ecol. Evol.* **2020**, *32*, 6, 509-526 [https://doi.org/10.1080/03949370.2020.1769739]

20. Ricklefs, R.E.; & Fallon, S.M. Diversification and host switching in avian malaria parasites. *Proc. Biol. Sci.* **2002**, 269(1494), 885–892.[https://doi.org/10.1098/rspb.2001.1940]

21. Szymanski, M.M; & Lovette, I.J. High lineage diversity and host sharing of malarial parasites in a local avian assemblage. *J. Parasitol.* **2005**, *91*, 768–774. [https://doi.org/10.1645/GE-417R1.1] [https://pubmed.ncbi.nlm.nih.gov/17089742/]

22. Pagenkopp, K.M.; Klicka, J.; Durrant, K.L.; Garvin, J.C.; Fleischer, R.C. Geographic variation in malarial parasite lineages in the Common Yellowthroat (*Geothlypis trichas*). *Conserv. Genet.* **2008**, *9*, 1577-1588. [https://doi.org/10.1007/s10592-007-9497-6]

23. Oakgrove, K.S.; Harrigan, R.J.; Loiseau, C.; Guers, S.; Seppi, B.; Sehgal, R.N. Distribution, diversity and drivers of blood-borne parasite co-infections in Alaskan bird populations. *Int. J. Parasitol.* **2014**, *44*, 717–727. [https://doi.org/10.1016/j.ijpara.2014.04.011]

24. Walther, E.L.; Carlson, J.S.; Cornel, A. *et al.* First molecular study of prevalence and diversity of avian haemosporidia in a Central California songbird community. *J. Ornithol*. **2016**, *157*, 549–564 [https://doi.org/10.1007/s10336-015-1301-7]

25. Smith, J.D.; Gill, S.A.; Baker, K.M.; Vonhof, M.J. Prevalence and diversity of avian Haemosporida infecting songbirds in southwest Michigan. *Parasitol. Res.* **2018**, *117*, 471–489. [https://doi.org/10.1007/s00436-017-5724-3] [https://pubmed.ncbi.nlm.nih.gov/29282527/]

26. Cozzarolo, C.S.; Jenkins, T.; Toews, D.; Brelsford, A.; Christe, P. Prevalence and diversity of haemosporidian parasites in the yellow-rumped warbler hybrid zone. *Ecol. Evol.* **2018**, *8*, 9834–9847. [https://doi.org/10.1002/ece3.4469] [http://www.ncbi.nlm.nih.gov/pmc/articles/pmc6202724/]

27. Martinsen, E.S.; Waite, J.L.; Schall, J.J. Morphologically defined subgenera of *Plasmodium* from avian hosts: test of monophyly by phylogenetic analysis of two mitochondrial genes. *Parasitology*. **2007**, *134*, 483–490. [https://doi.org/10.1017/S0031182006001922] [https://pubmed.ncbi.nlm.nih.gov/17147839/]

28. González, A.D.; Lotta, I.A.; García, L.F. Moncada, L.I.; Matta, N.E. Avian haemosporidians from Neotropical highlands: Evidence from morphological and molecular data. *Parasitol. Int.* **2015**, *64*, 48–59. [https://doi.org/10.1016/j.parint.2015.01.007] [https://pubmed.ncbi.nlm.nih.gov/25638289/]

29. Outlaw, D.C.; Ricklefs, R. E. On the phylogenetic relationships of haemosporidian parasites from raptorial birds (Falconiformes and Strigiformes). *J. Parasitol.* **2009**, *95*, 1171–1176. [https://doi.org/10.1645/GE-1982.1]

30. Sarquis-Adamson, Y.; & MacDougall-Shackleton, E.A. Song sparrows *Melospiza melodia* have a home-field advantage in defending against sympatric malarial parasites. *R. Soc. Open Sci.* **2016**, *3*, 160216. [https://doi.org/10.1098/rsos.160216] [http://www.ncbi.nlm.nih.gov/pmc/articles/pmc5108946/]

31. Boyd, R.J.; Kelly, T.R.; MacDougall-Shackleton, S.A.; MacDougall-Shackleton, E.A. Alternative reproductive strategies in white-throated sparrows are associated with differences in parasite load following experimental infection. *Biol. Lett*. **2018**, *14*, 20180194. [https://doi.org/10.1098/rsbl.2018.0194] [http://www.ncbi.nlm.nih.gov/pmc/articles/pmc6083222/]

32. Mijares, A.; Rosales, R.; Silva-Iturriza, A. Hemosporidian parasites in forest birds from Venezuela: genetic lineage analyses. *Avian Dis.* **2012**, *56*, 583–588. [https://doi.org/10.1637/10058-011312-ResNote.1] [https://pubmed.ncbi.nlm.nih.gov/23050478/]

33. Loiseau, C.; Harrigan, R.J.; Cornel, A.J.; Guers, S.L.; Dodge, M.; Marzec, T.; Carlson, J.S.; Seppi, B.; Sehgal, R.N. First evidence and predictions of *Plasmodium* transmission in Alaskan bird populations. *PLoS One*. **2012**, *7,* e44729. [https://doi.org/10.1371/journal.pone.0044729] [http://www.ncbi.nlm.nih.gov/pmc/articles/pmc3446979/]

34. Bosholn, M.; Fecchio, A.; Silveira, P.; Braga, É.M. and Anciães, M. Effects of avian malaria on male behaviour and female visitation in lekking blue-crowned manakins. *J. Avian Biol.* **2016**, *47*, 457-465. [https://doi.org/10.1111/jav.00864]
35. Fecchio, A.; Ribeiro, R.M.; Ferreira, F.C.; de Angeli Dutra, D.; Tolesano-Pascoli, G.; Alquezar, R.D.; Khan, A.U.; Pichorim, M.; Moreira, P.A.; Costa-Nascimento, M.J.; Monteiro, E.F.; Mathias, B.S.; Guimarães, L.O.; Simões, R.F.; Braga, É.M.; Kirchgatter, K.; Dias, R.I. Higher infection probability of haemosporidian parasites in Blue-black Grassquits (*Volatinia jacarina*) inhabiting native vegetation across Brazil. *Parasitol. Int.* **2021**, *80*, 102204. [https://doi.org/10.1016/j.parint.2020.102204]
36. Vanstreels, R.; Dutra, D.A.; Santos, A.P.; Hurtado, R.; Egert, L.; Braga, É.M. First report of avian malaria in a Manx shearwater (*Puffinus puffinus*). *Parasitol. Int.* **2020**, *78*, 102148. [https://doi.org/10.1016/j.parint.2020.102148] [https://pubmed.ncbi.nlm.nih.gov/32464257/]
37. Beadell, J.S.; Ishtiaq, F.; Covas, R.; Melo, M.; Warren, B.H.; Atkinson, C.T.; Bensch, S.; Graves, G.R.; Jhala, Y.V.; Peirce, M.A.; Rahmani, A.R.; Fonseca, D. M., Fleischer, R.C. Global phylogeographic limits of Hawaii's avian malaria. *Proc. Biol Sci.* **2006**, *273*, 2935–2944. [https://doi.org/10.1098/rspb.2006.3671] [http://www.ncbi.nlm.nih.gov/pmc/articles/pmc1639517/]
38. Spottiswoode, N.; Bartlett, S.L.; Conley, K.J.; Seimon, T.A.; Griffin, D.O.; Sykes, J.M.; Analysis of *Plasmodium* Lineages identified in captive penguins (*Sphenisciformes* spp.), eiders (*Somateria* spp.), and inca terns (*Larosterna Inca*) in a North American zoological collection. *J. Zoo Wildl. Med.* **2020**, *51*, 140–149. [https://doi.org/10.1638/2019-0078] [https://pubmed.ncbi.nlm.nih.gov/32212557/]
39. Doussang, D.; González-Acuña, D.; Torres-Fuentes, L. G.; Lougheed, S. C.; Clemente-Carvalho, R. B.; Greene, K. C.; Vianna, J. A. Spatial distribution, prevalence and diversity of haemosporidians in the rufous-collared sparrow, *Zonotrichia capensis*. *Parasit. Vectors.* **2019**, *12*, 2. [https://doi.org/10.1186/s13071-018-3243-4] [https://pubmed.ncbi.nlm.nih.gov/30606248/]
40. Merino, S.; Moreno, J.; Vásquez, R.A.; Martínez, J.; Sánchez-Monsálvez, I.; Estades, C.F.; Ippi, S.; Sabat, P.; Rozzi, R.; Mcgehee, S. Haematozoa in forest birds from southern Chile: Latitudinal gradients in prevalence and parasite lineage richness. *Austral. Ecol.* **2008**, *33*, 329-340. [https://doi.org/10.1111/j.1442-9993.2008.01820.x]
41. Galen, S.C.; and Witt, C.C. Diverse avian malaria and other haemosporidian parasites in Andean house wrens: evidence for regional co-diversification by host-switching. *J. Avian Biol.* **2014**, *45*, 374-386. [https://doi.org/10.1111/jav.00375]
42. Beadell, J.S.; and Fleischer, R.C. A restriction enzyme-based assay to distinguish between avian hemosporidians. *J. Parasitol.* **2005**, *91*, 683–685. [https://doi.org/10.1645/GE-3412RN] [https://pubmed.ncbi.nlm.nih.gov/16108566/]
43. Kimura, M.; Dhondt, A. A.; Irby J, L. Phylogeographic structuring of *Plasmodium* lineages across the North American range of the house finch (*Carpodacus Mexicanus*). *J. Parasitol.* **2006**, *92*, 1043–1049. [https://doi.org/10.1645/GE-639R.1] [https://pubmed.ncbi.nlm.nih.gov/17152948/]
44. Ishak, H.D.; Dumbacher, J.P.; Anderson, N.L.; Keane, J.J.; Valkiūnas, G.; Haig, S.M.; Tell, L.A.; Sehgal, R.N. Blood parasites in owls with conservation implications for the Spotted Owl (*Strix occidentalis*). *PloS One.* **2008**, *3*, e2304. [https://doi.org/10.1371/journal.pone.0002304]
45. Martinsen, E.S.; Perkins, S.L.; Schall, J.J. A three-genome phylogeny of malaria parasites (*Plasmodium* and closely related genera): evolution of life-history traits and host switches. *Mol. Phylogenet. Evol*. **2008**, *47*, 261–273. [https://doi.org/10.1016/j.ympev.2007.11.012] [https://pubmed.ncbi.nlm.nih.gov/18248741/]
46. Martinsen, E.S.; Sidor, I.F.; Flint, S.; Cooley, J.; Pokras, M.A. Documentation of Malaria Parasite (*Plasmodium* spp.) Infection and Associated Mortality in a Common Loon (*Gavia immer*). *J. Wildl Dis*. **2017**, *53*, 859–863. [https://doi.org/10.7589/2016-08-195] [https://pubmed.ncbi.nlm.nih.gov/28665230/]
47. Galen, S.C.; Nunes, R.; Sweet, P.R.; *et al.* Integrating coalescent species delimitation with analysis of host specificity reveals extensive cryptic diversity despite minimal mitochondrial divergence in the malaria parasite genus *Leucocytozoon*. *BMC Evol. Biol.* **2018**, *18*, 128 [https://doi.org/10.1186/s12862-018-1242-x]
48. Turcotte, A.; Bélisle, M.; Pelletier, F.; Garant, D. Environmental determinants of haemosporidian parasite prevalence in a declining population of Tree swallows. *Parasitology*. **2018**, *145*, 961–970. [https://doi.org/10.1017/S0031182017002128] [https://pubmed.ncbi.nlm.nih.gov/29166965/]
49. Pulgarín-R, P.C.; Gómez, C.; Bayly, N.J.; et al. Migratory birds as vehicles for parasite dispersal? Infection by avian haemosporidians over the year and throughout the range of a long-distance migrant. *J. Biogeogr*. **2019**, *46*, 83– 96. [https://doi.org/10.1111/jbi.13453]
50. Bueno, M.G.; Lopez, R.P.; de Menezes, R.M.; Costa-Nascimento, M., Lima, G.F.; Araújo, R.A.; Guida, F.J., Kirchgatter, K. Identification of Plasmodium relictum causing mortality in penguins (*Spheniscus magellanicus)* from São Paulo Zoo, Brazil. *Vet. Parasitol*. **2010**, *173*, 123–127. [https://doi.org/10.1016/j.vetpar.2010.06.026] [https://pubmed.ncbi.nlm.nih.gov/20638795/]
51. Tostes, R.; Dias, R.; de Oliveira, L.; Senra, M.; Massard, C.L.; & D'Agosto, M. Molecular and Morphological Characterization of a Brazilian Lineage of *Plasmodium* (Novyella) Unalis in *Turdus* Spp. (Passeriformes) of the Atlantic Forest, with Remarks on New Hosts and High Genetic Variation. *J. Parasitol.* **2018**, *104*, 70–78. [https://doi.org/10.1645/16-189] [https://pubmed.ncbi.nlm.nih.gov/28930498/]

52. Vanstreels, R.; Dutra, D.A.; Ferreira-Junior, F.C.; Hurtado, R.; Egert, L.; Mayorga, L.; Bhering, R.; Braga, É.M.; Catão-Dias, J.L. Epidemiology, hematology, and unusual morphological characteristics of *Plasmodium* during an avian malaria outbreak in penguins in Brazil. *Parasitol. Res*. **2019**, *118*, 3497–3508. [https://doi.org/10.1007/s00436-019-06459-8] [https://pubmed.ncbi.nlm.nih.gov/31720833/]
53. Marzal, A.; García-Longoria, L.; Cárdenas Callirgos, J.M.; Sehgal, R.N.M. Invasive avian malaria as an emerging parasitic disease in native birds of Peru. *Biological Invasions.* **2014**, *17*, 39-45. [https://doi.org/10.1007/s10530-014-0718-x]
54. Dodge, M.; Guers, S.L.; Sekercioğlu, Ç. H.; Sehgal, R.N. North American transmission of hemosporidian parasites in the Swainson's thrush (*Catharus ustulatus*), a migratory songbird. *J. Parasitol*. **2013**, *99*, 548–553. [https://doi.org/10.1645/GE-3134.1] [https://pubmed.ncbi.nlm.nih.gov/23030456/]
55. Martínez, J.; Vásquez, R.; Venegas, C.; Merino, S. Molecular characterisation of haemoparasites in forest birds from Robinson Crusoe Island: Is the Austral Thrush a potential threat to endemic birds? *Bird Conserv. Int.* **2015**, *25*, 139-152. [https://doi.org/10.1017/S0959270914000227]
56. Jones, M.R.; Cheviron, Z.A.; Carling, M.D. Spatial patterns of avian malaria prevalence in *Zonotrichia capensis* on the western slope of the Peruvian Andes. *J. Parasitol.* **2013**, *99*, 903–905. [https://doi.org/10.1645/12-147.1] [https://pubmed.ncbi.nlm.nih.gov/23517316/]
57. Mantilla, J.S.; González, A.D.; Lotta, I.A.; Moens, M.; Pacheco, M.A.; Escalante, A.A.; Valkiūnas, G.; Moncada, L.I.; Pérez-Tris, J.; Matta, N.E. *Haemoproteus erythrogravidus* n. sp. (Haemosporida, Haemoproteidae): Description and molecular characterization of a widespread blood parasite of birds in South America. *Acta Trop*. **2016**, *159*, 83–94. [https://doi.org/10.1016/j.actatropica.2016.02.025] [https://pubmed.ncbi.nlm.nih.gov/26995696/]
58. Cuevas, E.; Vianna, J.A.; Botero-Delgadillo, E.; Doussang, D.; González-Acuña, D.; Barroso, O.; Rozzi, R.; Vásquez, R.A.; Quirici, V. Latitudinal gradients of haemosporidian parasites: Prevalence, diversity and drivers of infection in the Thorn-tailed Rayadito (*Aphrastura spinicauda*). *Int. J. Parasitol. Parasites Wildl.* **2019**, *11*, 1–11. [https://doi.org/10.1016/j.ijppaw.2019.11.002] [http://www.ncbi.nlm.nih.gov/pmc/articles/pmc6920315/]
